# Therapeutic and preventive effects of astaxanthin in ischemic stroke

**DOI:** 10.3389/fnut.2024.1441062

**Published:** 2024-09-25

**Authors:** Xu Wang, Hongyan Li, Gaihua Wang, Ziqiao He, Xueting Cui, Feiyu Song, Jinhua Li, Lin Zhang

**Affiliations:** ^1^China-Japan Union Hospital, Jilin University, Changchun, Jilin, China; ^2^School of Public Health, Jilin University, Jilin, China; ^3^College of Traditional Chinese Medicine, Changchun University of Chinese Medicine, Jilin, China; ^4^Hospital of Stomatology, Jilin University, Changchun, Jilin, China; ^5^Jilin Connell Pharmaceutical Co., Ltd., Jilin, China

**Keywords:** astaxanthin, ischemic stroke, carotenoid, neuroprotective effect, parthanatos

## Abstract

Ischemic stroke poses a significant global health challenge with limited treatment options. Tissue plasminogen activator, the only effective medication, has strict restrictions, limiting its benefits only to a small number of patients. Astaxanthin, a natural carotenoid found in algae, shrimp, and crabs, has demonstrated promising neuroprotective properties in models of ischemic stroke. This article reviews the novel finding of neuroprotective impact of astaxanthin in ischemic stroke, highlighting its benefits in various protective mechanisms such as antioxidation, anti-inflammation, enhancement of DNA repair, anti-cell death, protection of blood–brain barrier, and promotion of neuronal survival. This analysis underscores the therapeutic and preventive potential of astaxanthin in ischemic stroke, positioning it as a prospective pharmaceutical agent against ischemic stroke.

## Graphical abstract

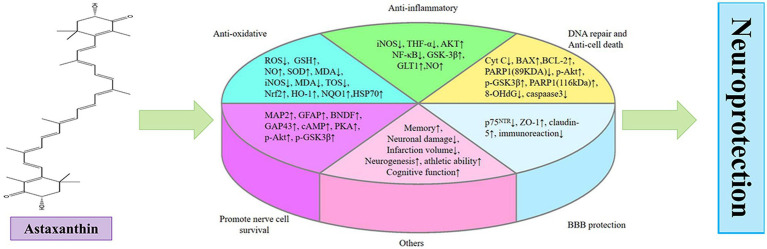

## 1 Introduction

Stroke encompasses both ischemic stroke (IS) and hemorrhagic stroke, with IS being the predominant form and responsible for approximately 85% of all strokes on a global scale (1), and the prevalence of IS is on the rise annually. The exact cause of IS is intricate and not well understood. Literatures indicated that the potential factors of IS consist of an overabundance of reactive oxygen species (ROS) (2), DNA damage, neuroinflammation (3), cell death, and blood–brain barrier (BBB) damage (3). Research has shown that medications that enhance blood flow to the brain, drugs that dissolve blood clots, and substances that protect the nervous system are successful in decreasing neurological impairments post cerebral ischemia. However, challenges persist in the practical implementation of these treatments (4). For instance, utilizing thrombolytic therapy proves to be a potent treatment option, however, its clinical application is hindered by stringent contraindications, a narrow treatment time window, and serious adverse reactions. Consequently, only a small number of patients experience its benefits (5). The prevailing belief is that ischemic injury to the brain is permanant and cannot be reversed. Hence, a focus on prevention and treatment of this condition is crucial, along with a deeper investigation into the etiology of IS and the creation of innovative pharmaceuticals.

The complex pathological mechanism of IS involves oxidative stress, which plays a crucial role in the progression of IS. Consequently, there is a growing interest in the development of potent antioxidants for treating IS (6). Astaxanthin (ATX) exhibits antioxidative potential that surpasses other carotenoids by a factor of 10 and outperforms α-tocopherol by 100–500 times in terms of activity (7, 8). ATX, a potent antioxidant produced by algae to combat external stressors, provides a protective shield against oxidative damage induced by UV radiation (9, 10). The shrimps grown in the absence of ATX will appear white and lack color (11). Although ATX plays a significant role in the coloration of shrimp bodies, its primary function in marine creatures is as an antioxidant. ATX enhances survival and growth, and boosts resistance to both physical and chemical stressors (12, 13). Based on studies of pharmacokinetics, ATX has the ability to pass through lipid membranes, enabling it to cross the BBB (14, 15).

The role of ATX in neurodegenerative diseases has been extensively reviewed (16). Currently, there has been no examination of the use of ATX for the treatment and prevention of IS. This article presents a novel review of the potential therapeutic benefits of ATX for IS, encompassing its abilities to combat oxidative stress, inflammation, apoptosis, and DNA damage, as well as providing protection to the BBB. The aim of this investigation is to establish a groundwork for utilizing ATX in the prevention and treatment of IS.

## 2 Mechanism of ATX in the prevention and treatment of cerebral ischemia

### 2.1 Improving the symptoms of ischemic stroke

ATX showed a potential in enhancing the conditions of IS. Numerous investigations have demonstrated that various treatments with ATX or prophylactic use can improve the infarct size in animal models with IS (11, 17, 18). The animals treated with ATX exhibited enhanced motor function and an improvement in neurological deficits due to the reduction in the size of the infarct (15, 17, 18). Additionally, ATX has demonstrated preventative and protective impacts on IS. These protective effects encompass vasodilation, enhanced blood circulation, and decreased thrombosis (11, 19, 20).

ATX may function by increasing nitric oxide (NO) levels, leading to vasodilation, increasing cerebral blood flow, and decreasing risk of thrombosis. Through the Phosphoinositide 3-kinase (PI3K)/protein kinase B (AKT) signaling pathway, ATX regulates phosphorylation of endothelial nitric oxide synthase to produce NO, thereby controlling vascular tone (20, 21). Additionally, memory loss induced by hippocampal damage following IS was ameliorated by ATX (19). The aforementioned protective benefits are directly linked to ATX’s enhancement of neuronal cell viability post IS (22). The mechanisms involved anti-oxidative stress, anti-inflammation, anti-apoptosis, anti-parthanatos, promotion of neuronal cell survival, enhancement of DNA repair, and BBB protection. The specific information is shown in Table 1.

**Table 1:** Details of included studies and results of astaxanthin in the treatment of IS.

References	Year	Species	Dose	Model	IS time	Evidence
17	2022	Male Wistar	25, 45, and 65 mg/kg	MCAO	60 min	Neurological deficit score↓, stroke volume↓, MDA↓, TOS↓, glutathione level↑, score of gait disturbance test↓, GLT1↑, NF-κB↑, TNF-α↑, caspase3↓, Bax↓, Bcl2↑
19	2005	Male Wistar	50 mg/kg	BCCAO	20 min	Arterial blood pressure↓, the latency of escaping onto the platform in the Morris water maze learning performance test↓
20	2011	Male RAT	300/600 mg/kg	Photochemical cerebral thrombus animal model	24 h	Arterial blood pressure↓, urinary 8-OHdG levels↓, Thrombogenesis↓, NO↑
18	2017	Male SD rat	5 mg/10 mg/kg	MCAO	2 h	Neurological deficit score↓, stroke volume↓, MDA↓, SOD↑, MAP-2↑, GFAP↑, BNDF↑, GAP43↑, Bax↓, Bcl2↑, Nrf2↑, HO-1↑, NQO1↑
11	2009	MaleSD rat	20 mL of 0.1 mM	MCAO	24 h	Cerebral blood flow↑, apoptosis↓, MDA↓, Cyt C↓, stroke volume↓, hydrogen peroxide↓
55	2019	Male mice and bEnd.3 Cells	40 mg/80 mg/kg or 20 μM	BCCAO	60 min	p75NTR↓, ZO-1↑, claudin-5↑, apoptosis↓
29	2017	Male mice	10 mg/kg/day	BCCAO	28 d	MDA↓, GSH↑, SOD↑, Cyt C↓, caspase3↓, BAX↑, BCL-2↑
15	2019	SH-SY5Y	5, 10, 20, and 40 μmol/L	OGD	24 h	Athletic ability↑, GAP43↑, cAMP↑, PKA↑
21	2010	Male mice	30 mg/kg	MCAO	10 min	ROS↓, caspase3↓, Bax↑, Bcl-2↑, p-Akt↑, p-GSK3β↑, Nrf2↑, HO-1↑
18	2022	Male gerbils	100 mg/kg	MCAO	5 d	PARP1 (116KDa)↑, PARP1 (89KDA)↓, iNOS↓, HO-1↑, HSP70↑
22	2022	Male Wistar	25, 45, and 65 mg/kg	MCAO	60 min	Neuronal survival↑

### 2.2 Anti-oxidative effects

Oxidative stress occurs when there is an imbalance between the antioxidants and oxidants in the body, leading to an accumulation of free radicals. These ROS and reactive nitrogen species such as superoxide anion, hydrogen peroxide, NO, and peroxynitrite anion are not effectively scavenged due to a lack of antioxidants (23). Antioxidants primarily consist of superoxide dismutase (SOD), glutathione peroxidase, catalase, and so forth. The brain is particularly vulnerable to oxidative harm in contrast to other organs, due to its increased consumption of oxygen, lipid levels, and reduced antioxidant enzymes. Additionally, the brain’s high energy consumption and limited energy reserves rely entirely on a consistent provision of oxygen and glucose by the vascular system (24, 25).

**Figure 1:**
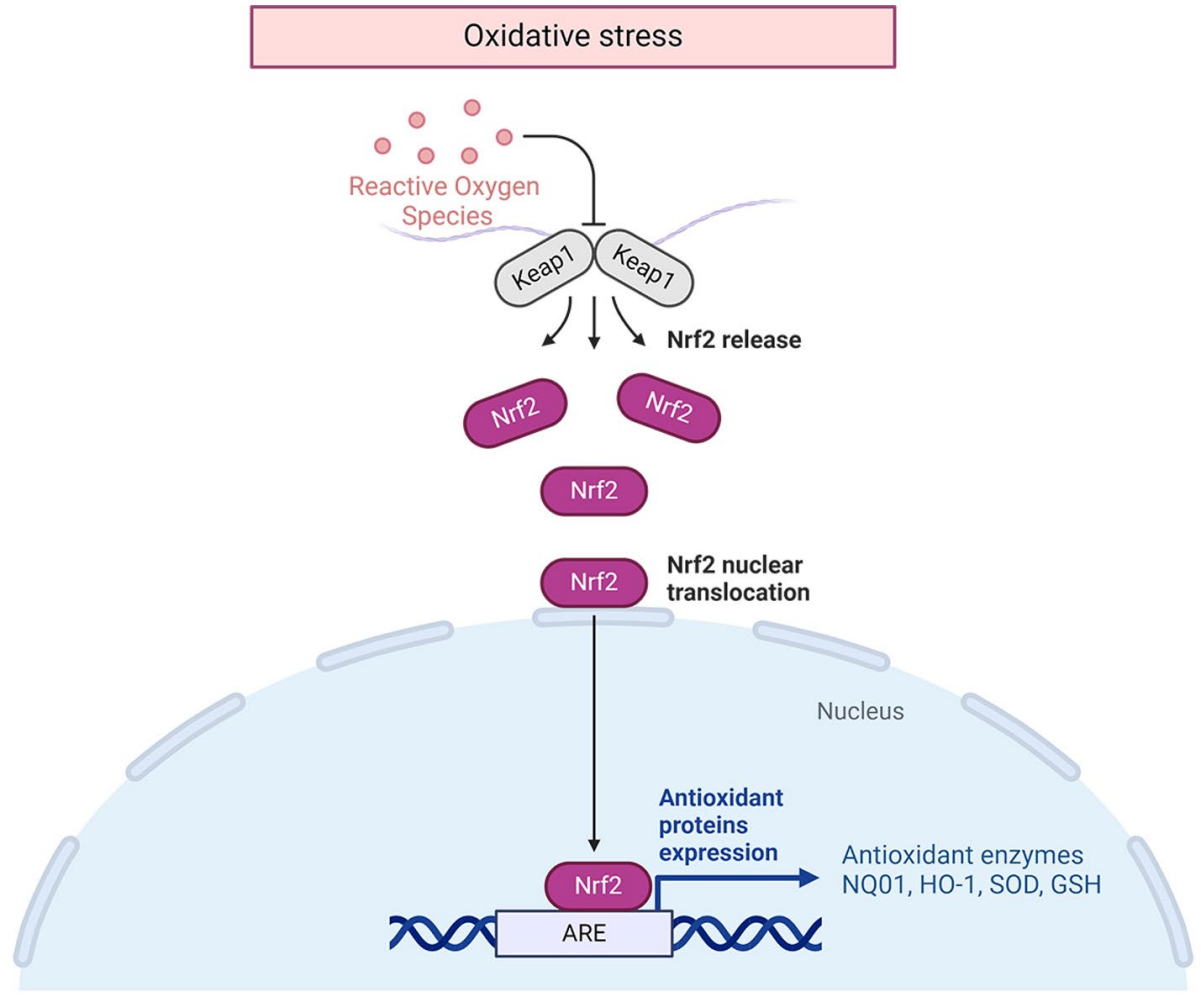
The molecular mechanisms of the antioxidative effects of ATX in IS.

Following an IS, the disruption of ion balance triggers an overproduction of free radicals by mitochondria, leading to neuronal cell demise. Consequently, the use of antioxidant emerges as a hopeful approach for tackling IS. ATX exhibits significant antioxidant properties, making it a viable candidate for managing this condition. An increasing body of experimental data supports the notion that oxidative stress is a central player in the development of cerebral ischemia. Abnormally high levels of ROS can distort lipid, protein, and DNA, ultimately causing cell fatality (26, 27). Malondialdehyde (MDA) is formed when polyunsaturated fatty acids undergo oxidation, making it a useful indicator of oxidative stress. SOD, a superoxide dismutase family member, transforms superoxide radicals within mitochondria into substances that are less harmful (28). Following administration of ATX to animals with IS, there was a noteworthy reduction in indicators of oxidative damage such as MDA, ROS, and total oxidation state (TOS), and a substantial increase in antioxidants like SOD, glutathione (GSH), and NO (11, 17, 18, 20, 21, 29). The molecular mechanisms of the antioxidative effects of ATX in IS are shown in Figure 1.

The specific molecular mechanism against oxidative stress is closely related to the nuclear factor erythroid 2-related factor (Nrf2)/Hemoxygenase 1 (HO-1) pathway (21). Nrf2 is a transcription factor that has been suggested as a regulator of defense against stress caused by oxidation (30). Additionally, Nrf2 is a crucial redox-sensitive transcription factor with a basic leucine zipper motif that is essential for controlling antioxidant genes. Once there is oxidative stress, Nrf2 is released from the complex of Nrf2/Kelch-like ECH-associated protein 1(Keap1), allowing it to move into the nucleus and activate the transcription of genes associated with antioxidative response, such as HO-1 (31). This pathway plays a crucial role in cellular defense against oxidative stress, acting as the primary mechanism for maintaining cellular homeostasis (32). Nrf2/HO-1 regulates gene expression through increasing coupling reaction species (33) and cellular antioxidant capacity, thus controlling the synthesis of proteins essential for detoxification and removal of ROS. HO1 acts as the primary enzyme in the breakdown of heme, leading to the production of carbon monoxide, biliverdin, and unbound iron (34). The degradation derivatives of heme exhibit immunomodulatory, anti-apoptotic, and vasodilatory attributes. Carbon monoxide, acting as a signaling molecule with vasodilatory impacts, functions in an anti-inflammatory and anti-apoptotic capacity (35). Biliverdin reductase converts biliverdin into bilirubin, a process that also helps in neutralizing hydroxyl radicals and superoxide anions. Additionally, it plays a role in reducing lipid peroxidation by decreasing levels of MDA (36). The liberation of iron at no cost is beneficial for the creation of heavy ferritin chains and the stimulation of ATPase Fe transporter at the membrane. This can decrease the quantity of iron available for free by enhancing the release of iron within cells, consequently minimizing cellular oxidative harm (37). Heme oxygenase can be categorized into three subtypes: HO-1, HO-2, and HO-3. It is noted that HO-1 is produced by nearly all cells during periods of stress (38). HO-1 is present at a low level in most mammalian tissues (39). The expression of HO-1 was significantly increased in hypoxic nerve cells treated with ATX compared to untreated hypoxic cells, enhancing the cells’ antioxidant capacity (21).

### 2.3 Anti-inflammatory effects

Following an IS, the ischemic lesion releases a multitude of inflammatory cytokines, such as tumor necrosis factor-α (TNF-α), interleukin-1β (IL-1β), and IL-6. These cytokines have the potential to induce nerve damage and enhance the expression of selectin and intercellular cell adhesion molecule-1 (ICAM-1), thus enhancing permeability of cerebral vascular endothelial cells. Moreover, inflammatory molecules can attract peripheral neutrophils, macrophages, and lymphocytes, leading to infiltration of white blood cells that further exacerbate inflammation, resulting in a destructive cycle of nerve damage and inflammation. Furthermore, activation of microglia and astrocytes occurs, with microglia shifting from an anti-inflammatory M2 state to a pro-inflammatory M1 state, intensifying neuroinflammation.

Post-treatment with ATX, there was a reduction in expression of glial glutamate transporter 1 (GLT1), leading to decreased glutamate accumulation and mitigated inflammation (17). Furthermore, following an IS, ATX decreased the inducible nitric oxide synthase (iNOS) expression, which is considered as the M1-type indicator of microglial proinflammation (21). The impact of ATX on expression of nuclear factor kappa-B (NF-κB) post IS remains a topic of debate. NF-κB, a transcription factor, serves as a pivotal controller of inflammation. Its activation is crucial for stimulating the transcription of various proinflammatory mediators related to innate immunity, including cytokines and growth factors (40). Activation of NF-κB can be triggered by various factors, such as inflammatory agents, TNF-α, IL-1β, hydrogen peroxide, and ROS. Activation of NF-κB occurs post-ischemia, and blocking the activation of NF-κB can help mitigate inflammatory damage (41). Antioxidants have the ability to prevent the activation of NF-κB. Nonetheless, a study discovered a notable rise in the expression of the NF-κB gene following the administration of ATX (17).

**Figure 2:**
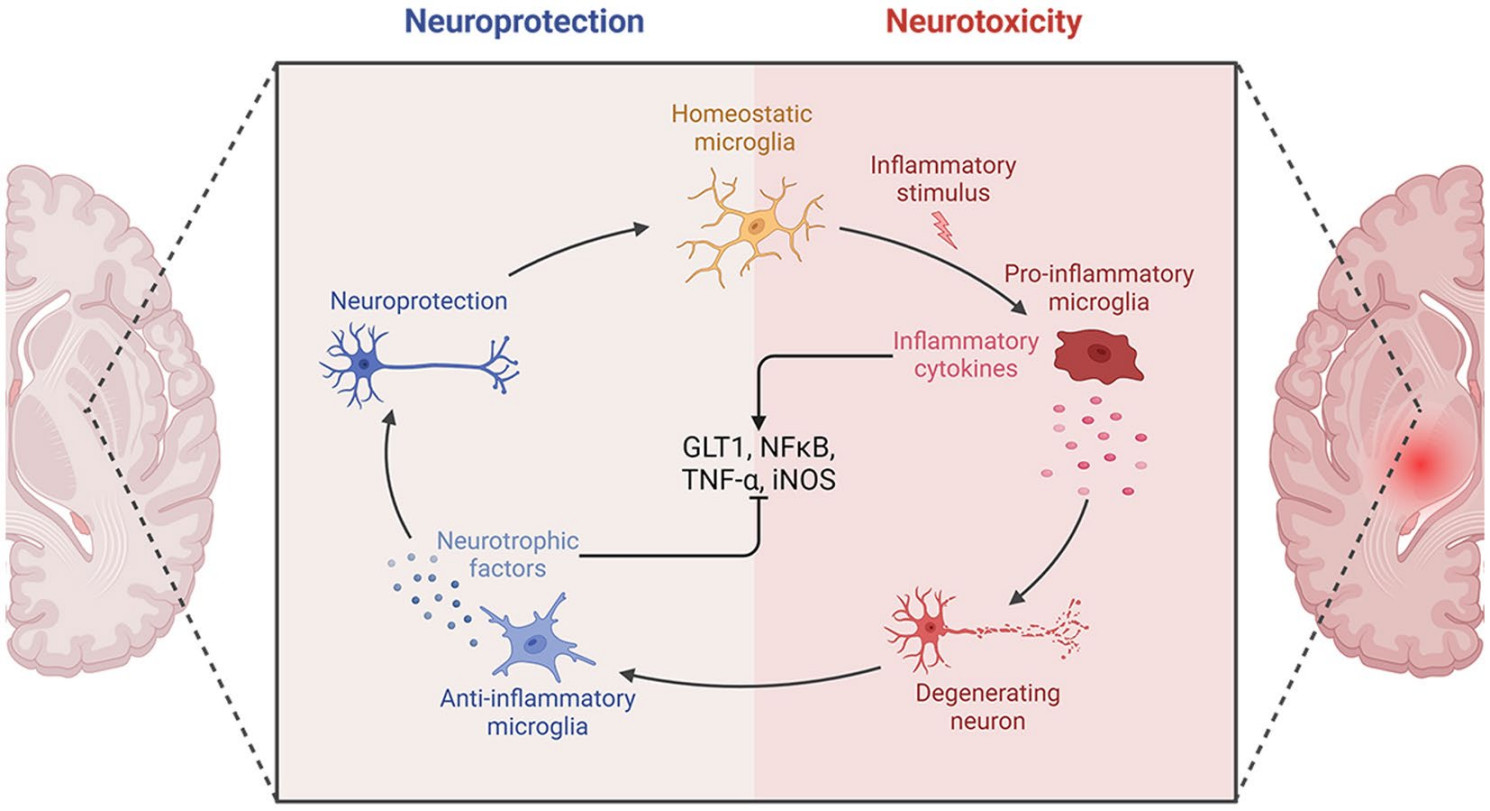
The molecular mechanisms of the anti-inflammatory effects of ATX in IS.

NF-κB plays a complex role in the inflammatory response, not always proinflammatory. It is associated with both the beginning and end of inflammation, as well as the promotion of apoptosis in leukocytes. One of the key factors affecting the inflammatory or anti-inflammatory impacts of NF-κB is the timing of sample collection for testing. Research on rat model with middle cerebral artery occlusion (MCAO) has revealed that activation of NF-κB typically initiates between 2 to 4 h following ischemic events, with a subsequent decrease in expression levels. However, there is a subsequent upsurge in NF-κB levels in the days following the ischemic event. In this particular study, samples were collected 30 h post-ischemia (17). This may be responsible for the increased expression of NF-κB. The molecular mechanisms of the anti-inflammatory effects of ATX in IS are shown in Figure 2.

### 2.4 Enhancement of DNA repair

Oxidative stress is a critical factor in the development of IS. Following the initiation of IS, excessive ROS is generated, resulting in oxidative damage to DNA, a significant outcome of oxidative stress (42), and can lead to DNA double-strand breaks and finally lead to neuronal cell death through a variety of mechanisms (42). When DNA sustains damage, a cellular response mechanism known as the DNA damage response is triggered. This mechanism involves homologous recombination and non-homologous end-joining to effectively repair the damage (43, 44). Moreover, poly (ADP-ribose) polymerase 1 (PARP1), a key member of the superfamily of poly (ADP-ribose) polymerases, plays a crucial role in both DNA repair pathways and the regulation of cellular apoptosis (45). Elevated levels of PARP-1 following an IS initiate the movement of apoptosis-inducing factor (AIF) into the nucleus, leading to breakdown of chromatin and cell death independent of caspase activation via binding to the histone variant H2A histone family member X (H2AX) (46, 47). The PARP/AIF pathway mediates cell death in a caspase-independent manner, which has been termed parthanatos. Oxidative stress-induced neuronal DNA damage during ischemia is closely linked to parthanatos.

Following administration of ATX, there was a decrease in the level of 8-hydroxy-2’-deoxyguanosine (8-OHdG), a marker of DNA damage, and an increase in the expression of PARP1 (116 kDa) protein, which plays a role in facilitating DNA repair, in animals experiencing IS (18, 20). Following ATX administration, there was a notable enhancement in the mRNA expression level of NAD(P)H quinone oxidoreductase 1 (NQO1). NQO1 plays a key role in the maintenance of cellular DNA repair mechanisms. Moreover, the upregulation of heat shock protein 70 (Hsp70) was observed post ATX treatment. Hsp70, a prominent inducible heat shock protein with a molecular weight of 70 kDa, aids in preserving protein functionality amidst challenging environmental conditions. It was demonstrated that cells demonstrating elevated levels of Hsp70 exhibited notable resilience against DNA damage caused by hydrogen peroxide (48). The above evidence suggests that ATX has an enhancement of DNA repair and can promote cell survival.

### 2.5 Anti-cell death effects

Programmed cell death encompasses apoptosis and parthanatos, among other mechanisms. Acting as a sensor for DNA damage, approximately 90% of polyADP ribose is generated in reaction to oxidative stress or DNA damage (49). Following IS, both active and passive DNA harm occurs. Cerebral ischemia triggers DNA damage as a reaction. DNA endonucleases, recognized as endonuclease-mediated DNA damage, are responsible for active DNA damage. The primary focus of research in active DNA damage is on apoptotic DNA fragmentation, identified by DNA double-strand breaks (50). DNA fragmentation entails a series of cellular self-destructive steps that are frequently irreversible. The primary endonucleases responsible for DNA fragmentation are caspase-activated deoxyribonucleases and AIF (51). Recent research indicates that a minimum of two pathways leading to AIF release are engaged: one relying on B-cell lymphoma-2 (Bcl-2) family proteins like Bax and caspases, and the other relying on PARP-1 (52). The relocation of AIF from the mitochondria to the nucleus was recognized as a crucial phase in the process of PARP-1 induced cell demise (53).

**Figure 3:**
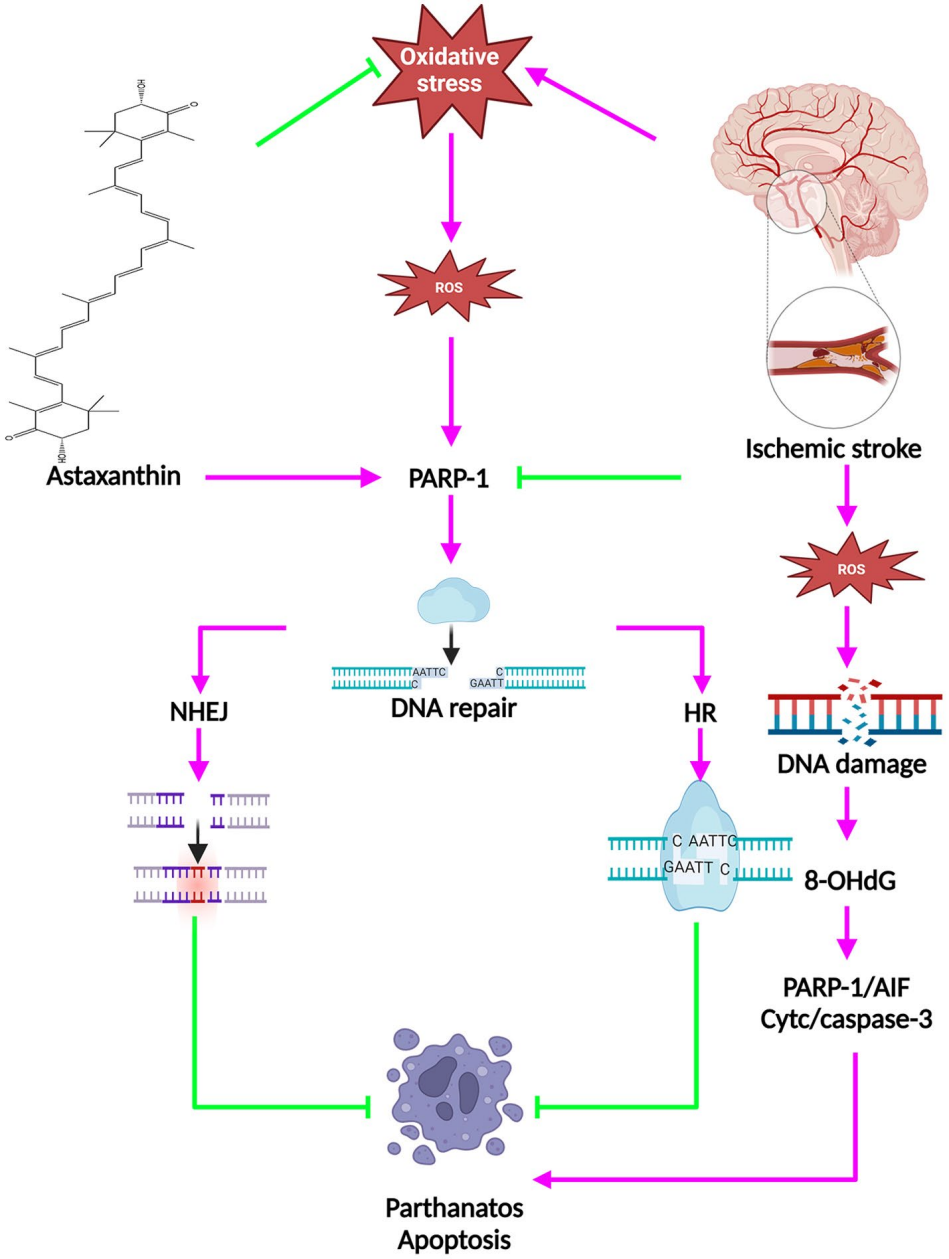
The molecular mechanisms of the anti-cell death effects of ATX in IS.

Researches suggested that inhibition of PARP1 has reduced migration of AIF from the mitochondria to the nucleus, leading to neuroprotection from IS-induced neuronal death (54). ATX elevated the levels of DNA repair protein PARP1 (116 kDa) and anti-apoptotic protein Bcl-2, while reducing the levels of parthanatos protein PARP1 (89 kDa) as well as pro-apoptotic proteins Bax, Caspase3, and cytochrome c (Cyt C) (11, 18, 21, 29, 55). The molecular mechanisms of the anti-cell death effects of ATX in IS are shown in Figure 3.

### 2.6 Protective effects on blood–brain barrier

The BBB plays a crucial role in shielding brain tissue from noxious substances and ensuring its protection (56). The probability of hemorrhagic transformation in individuals following both thrombolysis and mechanical extraction is approximately 10% (57). Additionally, approximately 40% of individuals diagnosed with IS and did not receive rtPA treatment were similarly identified as having hemorrhagic transformation, although the resulting effects were less significant compared to those following thrombolysis (58). The disruption of BBB’s integrity by IS mainly causes an increase in BBB permeability. Various factors contribute to BBB disruption, leading to severe outcomes such as heightened brain damage and mortality.

During the initial phase of a stroke, a range of injury-associated molecular patterns released from deceased and affected neurons, triggering glial activation, peripheral immune reaction, and heightened production of inflammatory mediators. This process hastens the breakdown of the BBB, exacerbates cerebral swelling, and disrupts microcirculation, leading to secondary brain damage. P75 neurotrophic receptor (p75NTR) is crucial in numerous physiological activities, such as controlling cell viability, forming scars, regulating energy consumption, and responding to hypoxia. By facilitating these functions, P75NTR enhances vital biological processes involved in tissue mending, metabolism, and degeneration of neurons (59). Knocking down of P75NTR reduced brain injury and BBB breakdown in mice subjected to MCAO. Conditional knockout of P75NTR greatly improved BBB integrity and reduced brain damage, along with enhancing neurological function post-stroke, especifically in astrocytes (60). Following treatment with ATX, the levels of P75NTR were reduced while protein expression of tight junction-related zona occludens 1 (ZO-1) and claudin-5 increased in animals with IS (55). These findings indicate that ATX may provide protection to the BBB by modulating the expression of P75NTR in astrocyte.

### 2.7 Promoting survival of nerve cells

Study have found that ATX can promote the survival of hypoxic nerve cells (22). Numerous factors influence axonal regeneration, including intricate intracellular and extracellular signal transduction mechanisms. Currently, the cyclic adenosine monophosphate (cAMP)/protein kinase A (PKA) signaling pathway is recognized as a crucial factor in regulating axon regrowth (61, 62). By activating the PKA-mediated signaling pathway, cAMP can uphold neurons’ robust growth, while also diminishing the negative impacts of neuron growth inhibitors on axon growth cones. This is achieved by influencing the molecular effects triggered by downstream gene transcription, ultimately facilitating axon regeneration (63, 64). Levels of cAMP, cAMP-response element binding protein (CREB), and PKA in the cortex of mice experiencing cerebral infarction showed an increase on 7 days ATX treatment, peaking at 14 days. This suggests that ATX triggers the cAMP/PKA/CREB signaling pathway by raising cAMP levels in brain tissue, leading to the enhanced axonal regeneration and improved motor function (15).

**Figure 4:**
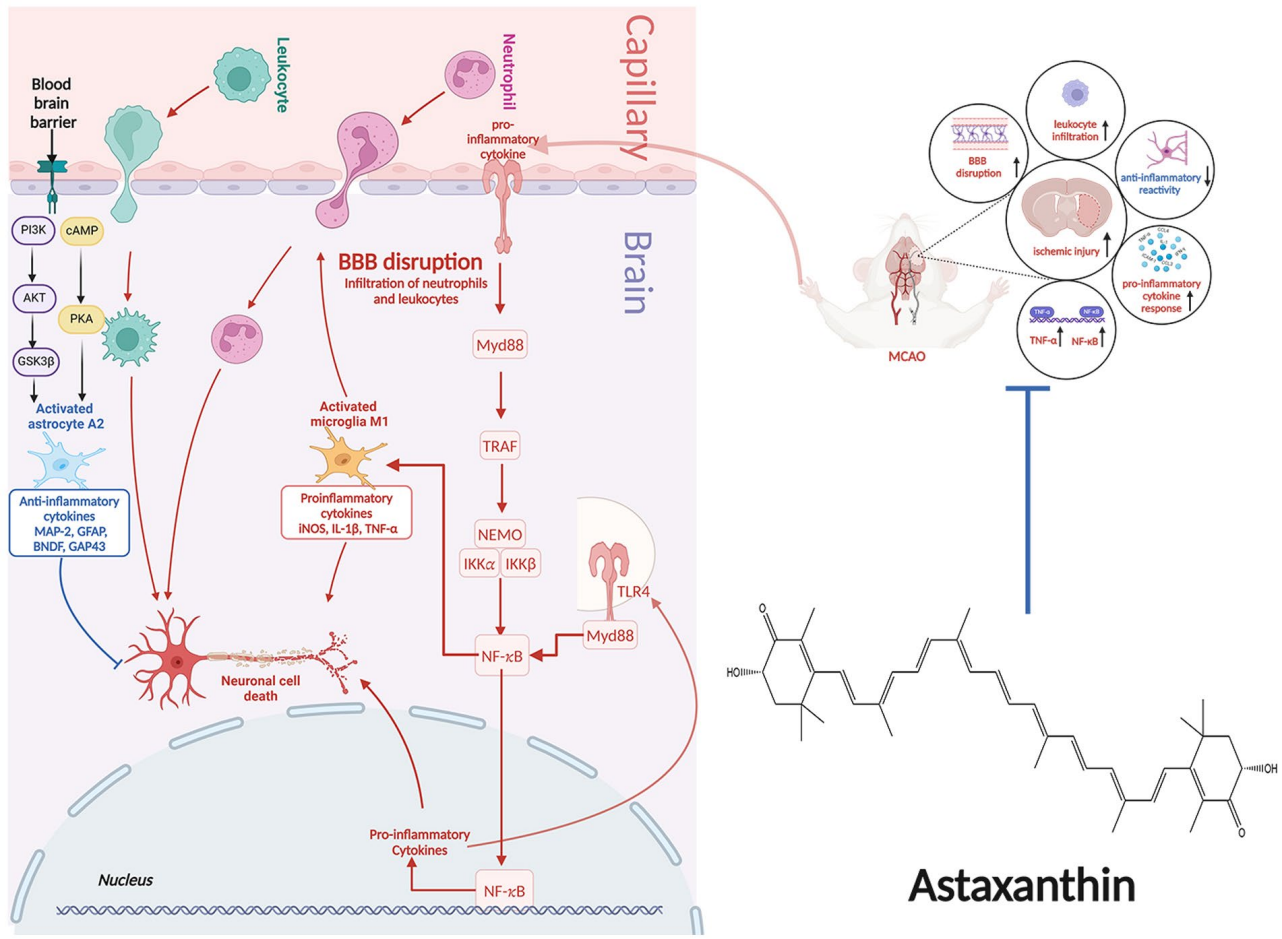
The molecular mechanisms of the promoting nerve cell survival effects of ATX in IS.

Growth-associated protein (GAP-43) is abundantly present during axon growth and differentiation, particularly at the tips of developing axons, providing guidance for axon elongation and serving as a dependable indicator of axon development (63, 64). The activation of GAP-43 upregulates the protein kinase pathway, leading to the enhancement of neurite formation, regeneration, and plasticity (65). Furthermore, another investigation discovered that ATX enhanced the regeneration of neurons through the upregulation of glial fibrillary acidic protein (GFAP), microtubule-associated protein2 (MAP-2), brain-derived neurotrophic factor (BDNF), and GAP-43 expression (18). GFAP is a signature intermediate filament in astrocytes that contributes to synaptic formation and axonal metabolic homeostasis in the central nervous system (66). Expression of the MAP-2 protein is also heightened, aiding in the stabilization of microtubule growth and playing a crucial role in nerve regeneration (67). An elevation in BDNF levels influences neurons within the central nervous system, assisting in maintaining the survival of current neurons while also encouraging the proliferation and specialization of fresh neurons (68). The molecular mechanisms of promoting nerve cell survival of ATX in IS are shown in Figure 4.

### 2.8 Limitations and future perspectives

The amount of ATX required to enhance blood flow and prevent blood clotting is excessive, at a dosage of 300 mg/kg (20). ATX is extremely potent, yet the amount of supplementation available in the market is limited. Research has indicated that only 6 mg of ATX per day is considered safe for adult consumption. In addition, the dosage of ATX for both prevention and treatment lacks clarity, along with uncertainties surrounding potential adverse reactions from excessive ATX usage and its interactions with other medications.

## 3 Conclusion

Stroke is a prominent reason for mortality and handicap globally. Despite extensive research, time, and funding devoted to discovering the optimum treatment, little progress has been made. Essentially, the solution may rest in nature, which could aid in improving prevention efforts. While medicine aims for a definitive cure, emphasis should also be placed on shielding individuals from stroke. This review inevitably has limitations, such as the small number of studies included. In addition, human clinical trials are required to evaluate the preventive and therapeutic impacts of ATX. At present, both *in vivo* and *in vitro* research on the chemoprotective properties of ATX is in its early stages. This situation presents an opportunity for further detailed investigations on ATX, encompassing a comprehensive evaluation of its metabolites, consideration of individual variability, examination of potential adverse effects and toxicity, and exploration of the long-term protective potentials of ATX.
